# Supplementary material for: Over-expression of ADAR1 in mice does not initiate or accelerate cancer formation in vivo
Source: NAR Cancer. 2023 Jun 1;5(2):zcad023. doi: 10.1093/narcan/zcad023 (PMC10233902; doi:10.1093/narcan/zcad023)
Supplement: zcad023_Supplemental_Files [file zcad023_supplemental_files.zip › Mendez Ruiz et al_NAR Can_Supp files_CW070323.pdf]

Supplementary Data for:

Overexpression of ADAR1 in mice does not initiate or accelerate cancer formation *in vivo*.

Shannon Mendez Ruiz[1,2], Alistair M Chalk[1,2], Ankita Goradia[1], Jacki Heraud-Farlow[1,2,†,*], Carl R Walkley[1,2, †,*].

[1] St Vincent's Institute of Medical Research, Fitzroy, Victoria, 3065, Australia.
[2] Department of Medicine, Eastern Hill Precinct, Melbourne Medical School, University of Melbourne, Fitzroy, Victoria, 3065, Australia.
† Joint authors

* To whom correspondence should be addressed. Tel: +61 3 9231 2480; Email: jhfarlow@svi.edu.au ; cwalkley@svi.edu.au

SUPPLEMENTARY DATA

Figures:

Supplemental Figure 1. Validation of Adar1 cDNAs. Expression of the indicated murine *Adar1* cDNA using lentivirus in the murine stromal cell line Kusa4b10 and western blot using **(A)** anti-Flag or **(B)** anti-ADAR1 antibody. Text in brackets indicates any modification to the *Adar1* cDNA. *Adar2* included as a control. **(C)** Graphical representation of the different protein products expected from each allele.

Supplementary Figure 2. Expression of GFP in adult tail fibroblasts derived from *Ubc*-CreER mice of the indicated genotype. Flow cytometry analysis of adult tail fibroblasts: Untreated **(A)** *Ubc*-CreER-ve *R26-Adar1*$^{ki/ki}$ and (B) *Ubc*-CreER$^{tg/+}$ *R26-Adar1*$^{ki/ki}$ cell lines. Panel **(C-H)** are tail fibroblasts treated with 400nM 4-hydroxy tamoxifen for 7 days: **(C)** *Ubc*-CreER-ve *R26-Adar1*$^{ki/ki}$ (Cre negative; from panel A); **(D)** *Ubc*-CreER$^{tg/+}$ *R26-Adar1*$^{ki/ki}$ (Cre+ve from panel B); **(E)** *Ubc*-CreER$^{tg/+}$ *R26-Adar1p150*$^{ki/ki}$; **(F)** *Ubc*-CreER$^{tg/+}$ *R26-Adar1-Za*$^{ki/ki}$; **(G)** *Ubc*-CreER$^{tg/+}$ *R26-Adar1p110*$^{ki/ki}$ and **(H)** *Ubc*-CreER$^{tg/+}$ *R26-Adar1E861A*$^{ki/ki}$.

Supplementary Figure 3. Editing of *Cdk13* recoding site in liver samples following 14 days of tamoxifen treatment. **(A)** IGV screen shot of *Cdk13* editing at the recoding p. Q103>R site; quantitation and statistical analysis of the editing frequency at the recoding site and average number of reads per sample for the site (expressed as mean +/- sem for each allele). ***P*<0.01, ****P*<0.001; Statistical comparisons using a two-way ANOVA with multiple comparisons correction using Prism software.

Supplementary Figure 4. Analysis of the contribution of the GFP-ve cells from the analysis of 18 month old animals. **(A)** The percentage contribution of peripheral blood GFP- cells (non-ADAR1 overexpressing cells) to each indicated lineage. **(B)** The percentage contribution of GFP- cells in the bone marrow to each indicated population. **(C)** The percentage contribution of GFP- cells in the bone marrow to the lineage-cKit+Sca1+ (LKS+) population and the long-term and short-term hematopoietic stem cell populations (contained within the LKS+ fraction). **(D)** The percentage contribution of GFP- cells in the bone marrow to the lineage-cKit+Sca1- (LKS-) population and the megakaryocyte progenitors (MkP), granulocyte macrophage progenitors (GMP), pre-GM, pre-Megakaryocyte erythroid progenitors (preMegE), pre colony forming unit erythroid (preCFU-E) and CFU-E populations (contained within the LKS- fraction). **(E)** Contribution of the GFP- cells to the indicated cell populations. **(F)** Contribution of the GFP- cells to the indicated cell populations. Each circle indicates an individual animal; **P*<0.05, ***P*<0.01, ****P*<0.001; *****P*<0.0001; Statistical comparisons using a two-way ANOVA with multiple comparisons correction using Prism software.

Supplementary Figure 5. FACS analysis of peripheral blood from *Ubc*-CreER R26-Adar1p110 mouse #130 at 18 months post tamoxifen. Analysis of haemolysed peripheral blood leukocytes in *Ubc*-CreER R26-Adar1p110 mouse #130 at 18 months post tamoxifen. Top panel shows gating of

leukocytes (FSC v SSC) and the GFP gating from the total leukocyte population. The lineage contribution from the GFP+ve and GFP-ve are then shown.

Supplementary Figure 6. Loss of *Trp53* in osteoblasts leads to an activation of the interferon stimulate gene expression program. (A) IGV screen shots of the *Trp53* (p53) locus at day 7, 14 and 21 of tamoxifen treatment. The region deleted by the $Trp53^{fl/fl}$ allele is indicated. The data show the read counts from RNA-seq. **(B)** The expression of the interferon stimulated gene set in each replicate at day 7, 14 and 21. Data expressed as counts per million.

Supplementary Figure 7. Overexpression of ADAR1 does not accelerate osteosarcoma formation. (A) Mean survival +/- standard deviation of each genotype in the KM plot in Figure 7B. **(B)** Kaplan Meier plot based on *Osx1*-Cre $p53^{fl/fl}$ genotype + the indicated *R26*-Adar1 allele. These can have either $pRb^{fl/+}$ or $pRb^{fl/fl}$ genotype. Number as indicated in the inset, no statistical difference. Median survival as determined by Prism KM analysis.

Supplementary Figure 8. Representative flow cytometry profiles and gating strategies for FACS analysis. Each page is labelled with the organ assessed and the antibodies and conjugates used.

Tables:

Supplementary Table 1. Genotyping primers used in this study.

Supplementary Table 2. Flow cytometry antibodies, dye conjugates, antibody clone and source used in this study.

Datasets:

Supplementary Dataset 1: Gene expression analysis of day 14 liver samples.

Supplementary Dataset 2: Editing analysis of day 14 liver samples.

Supplementary Dataset 3: Gene expression of osteoblasts.

Supplementary Dataset 4: Editing analysis of osteoblasts.

Supplementary Data are available at NAR online.

Supplemental Figure 1

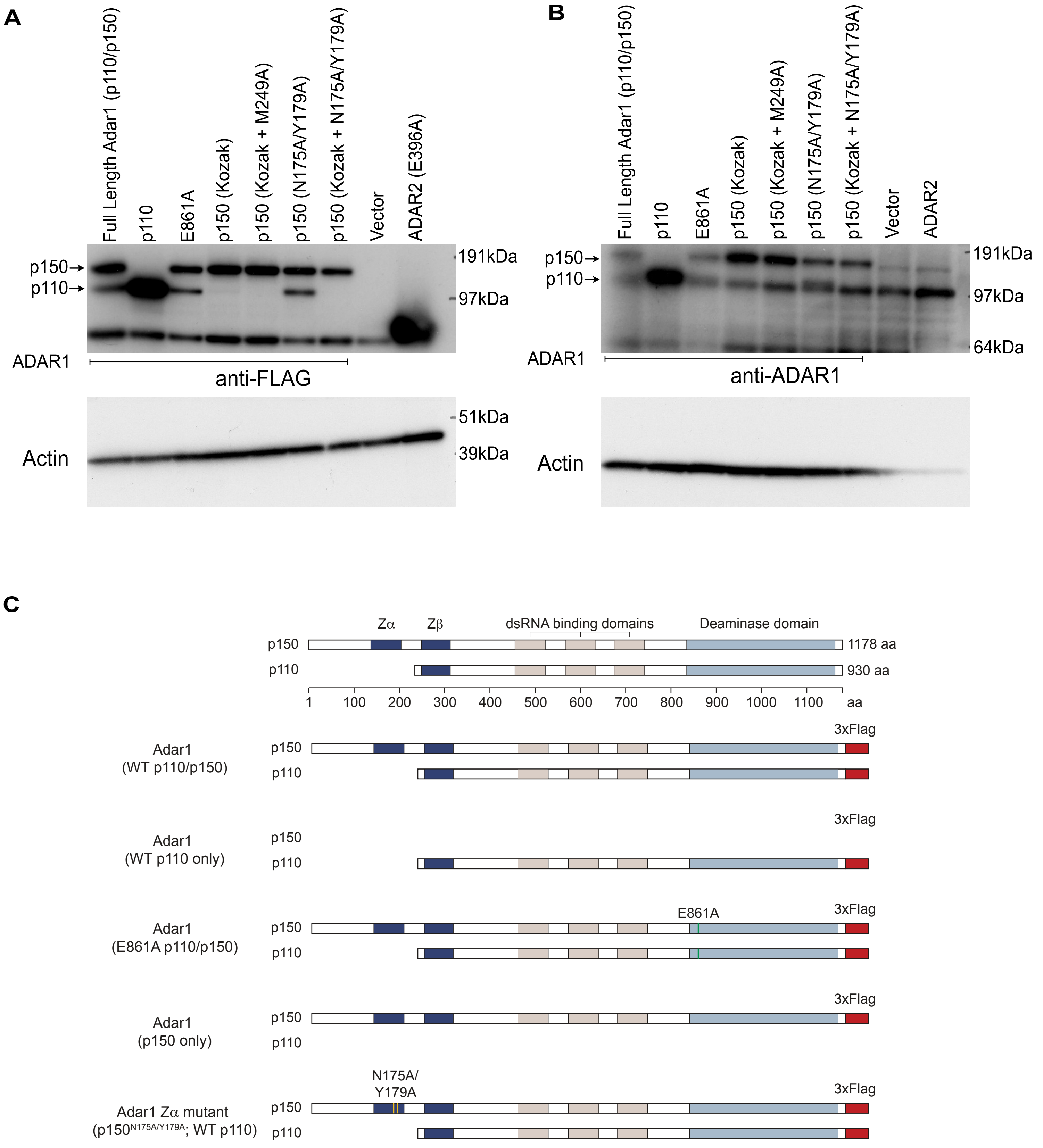

Supplemental Figure 2

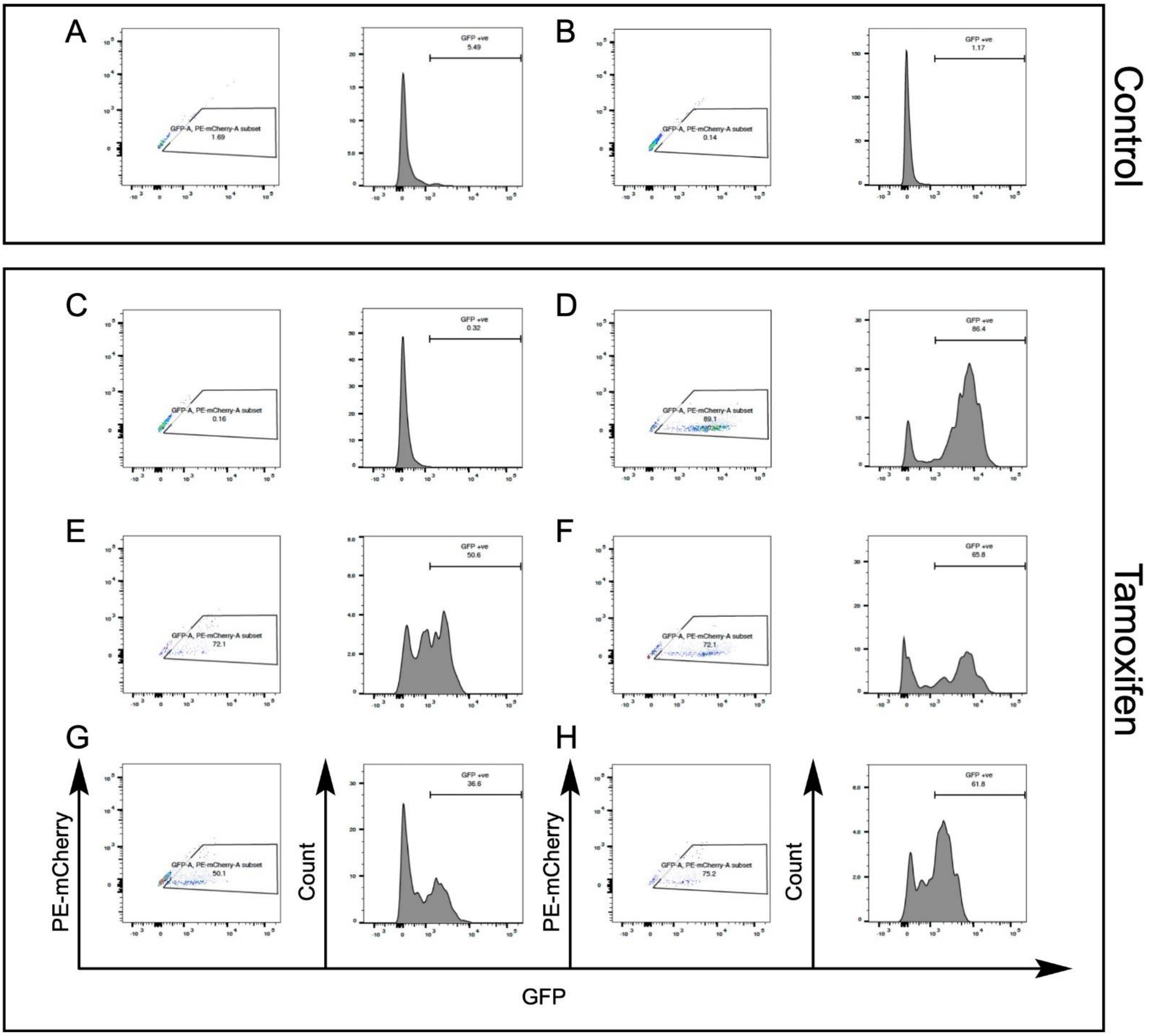

Supplemental Figure 3

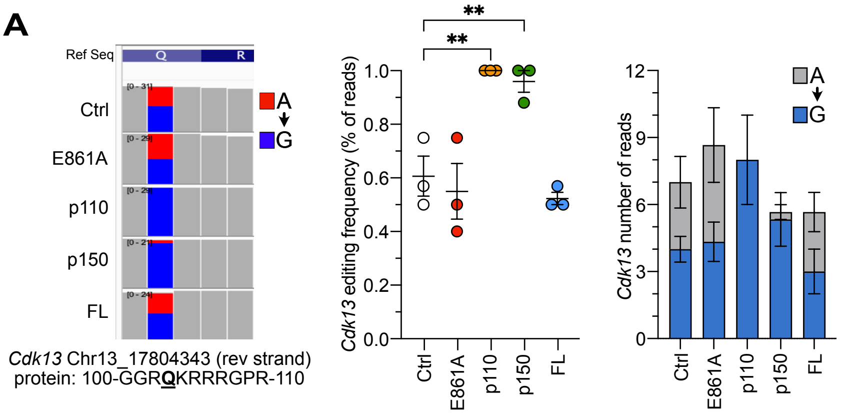

Supplemental Figure 4

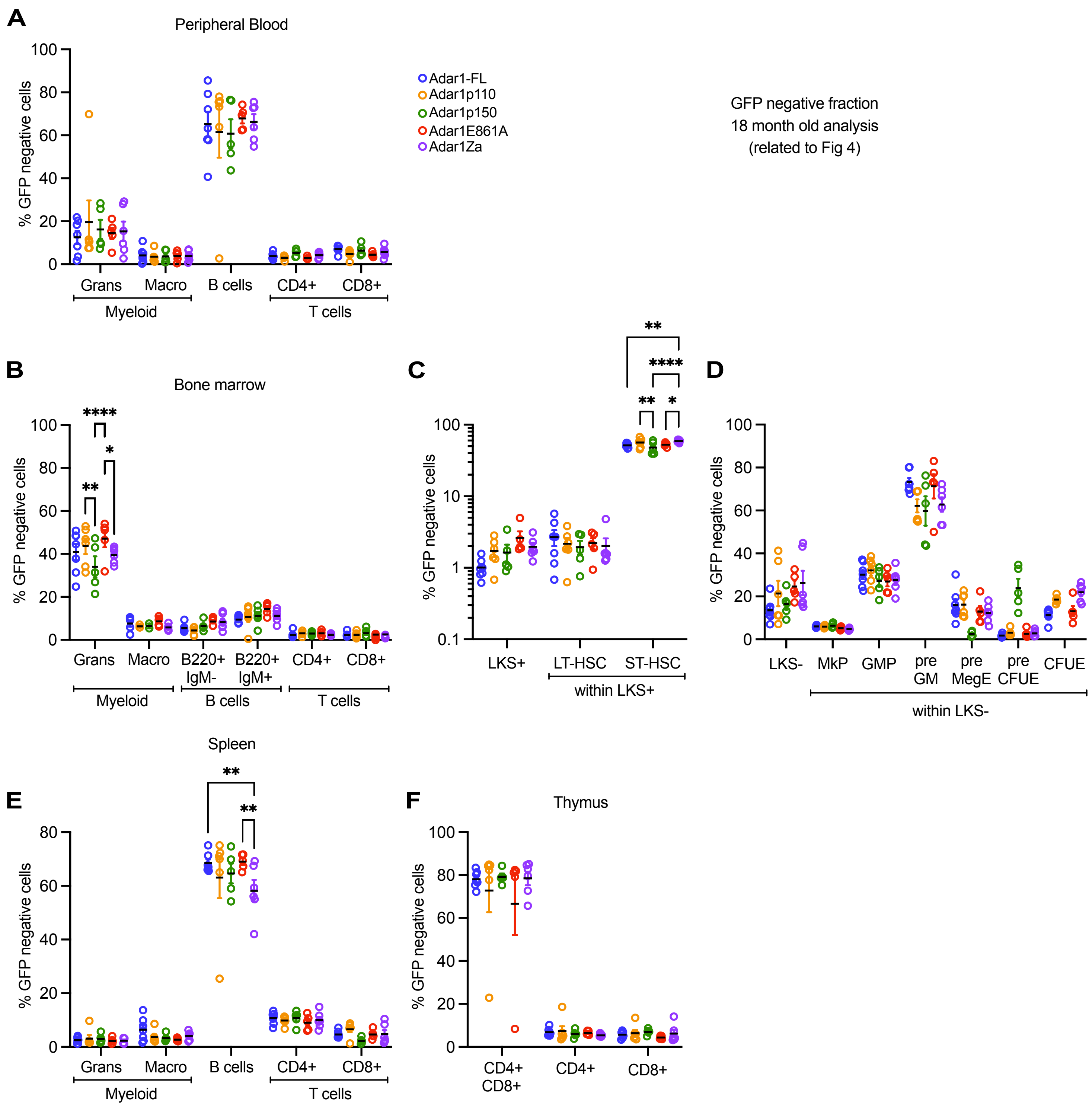

Supplemental Figure 5

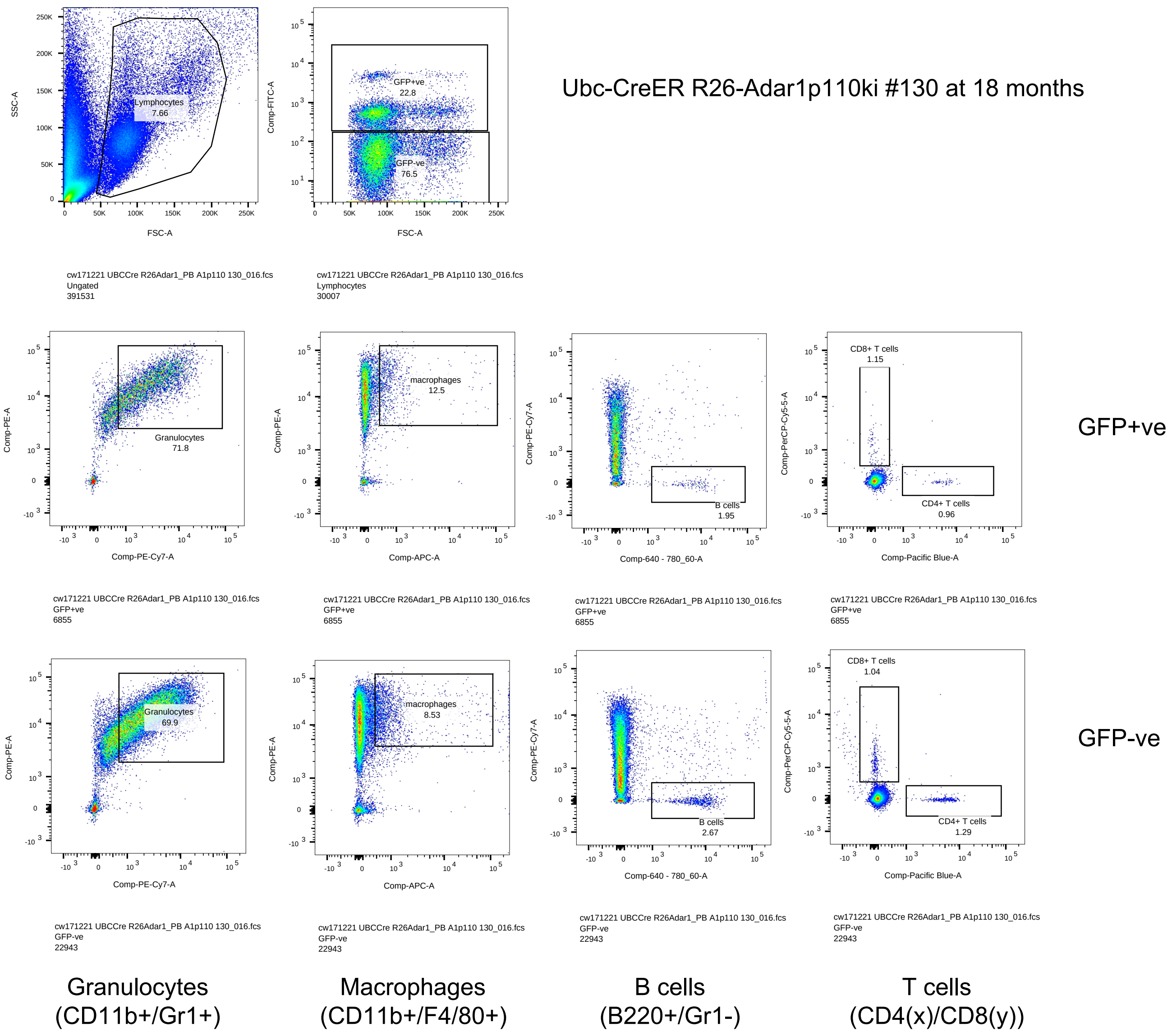

Supplemental Figure 6

A

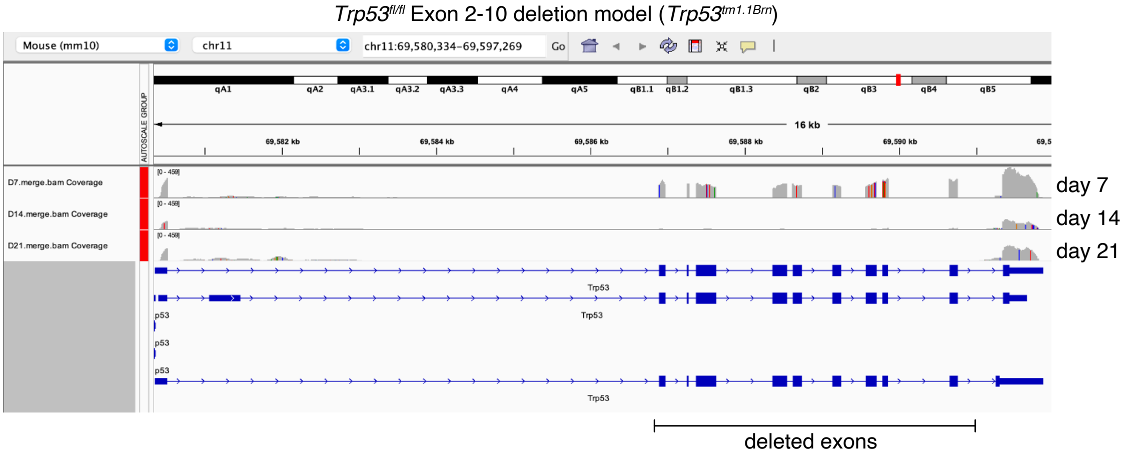

B

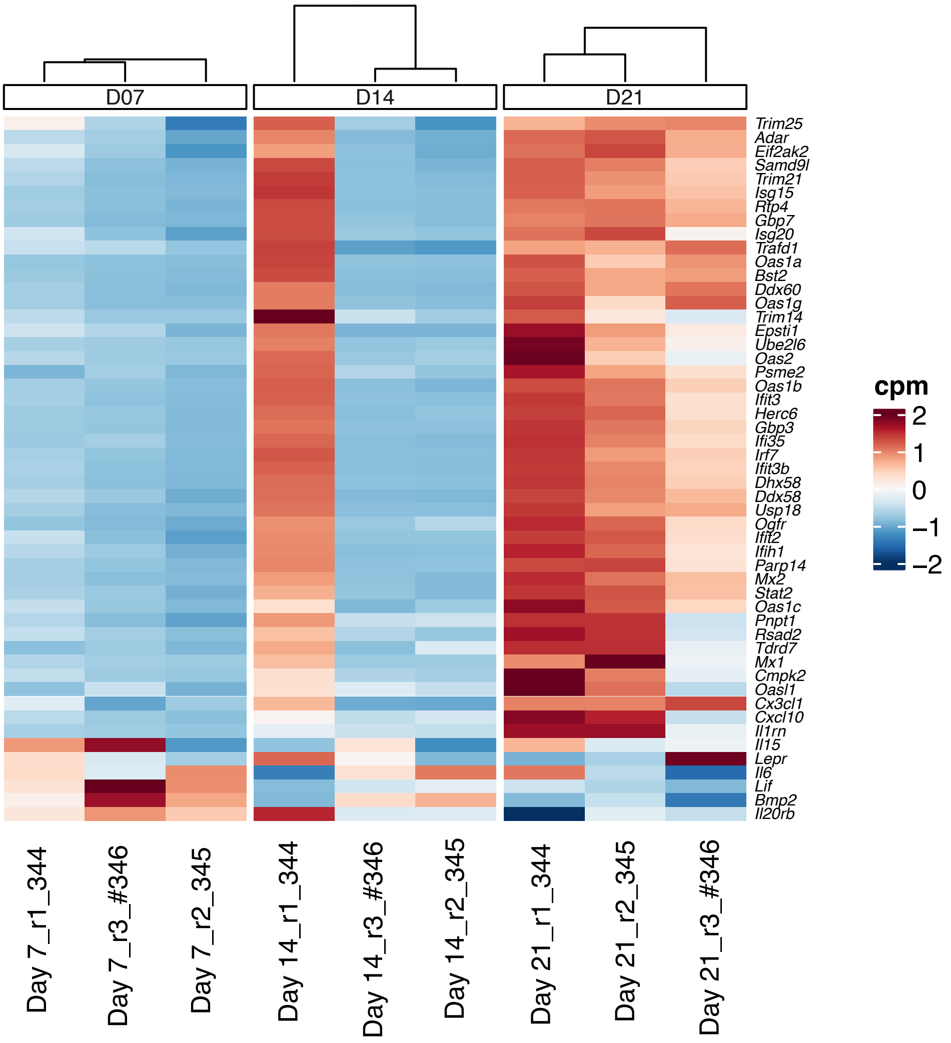

Interferon/Cytokine gene set

Supplemental Figure 7

A

	Mean +/- SD (Days)
Control	196.6 +/- 24.81
Adar1 FL	235.0 +/- 96.04
Adar1p150	204.1 +/- 76.20
Adar1p110	189.0 +/- 49.22
Adar1 E861A	214.2 +/- 52.50

B

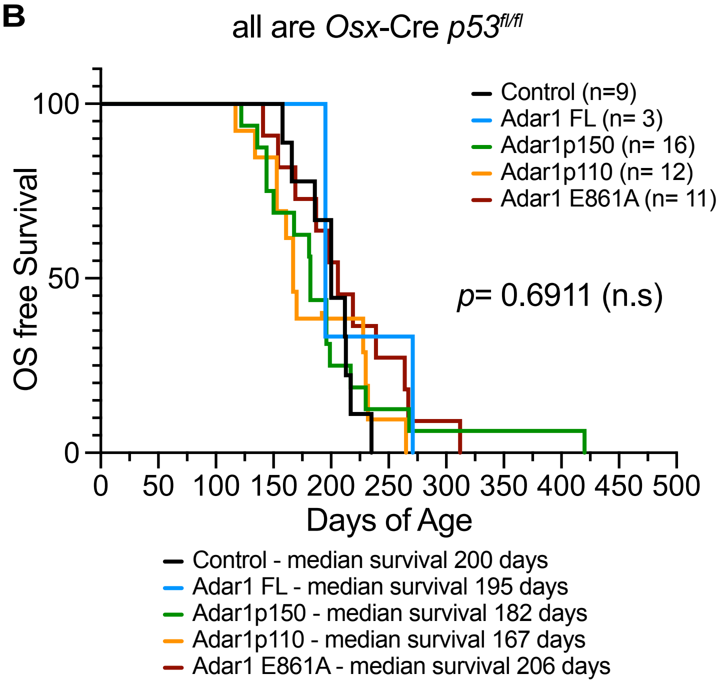

Supplemental Figure 8

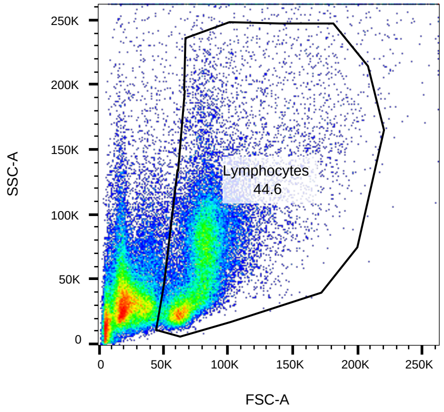

cw171221 UBCCre R26Adar1_BM M A1 Za 121_037.fcs
Ungated
67728

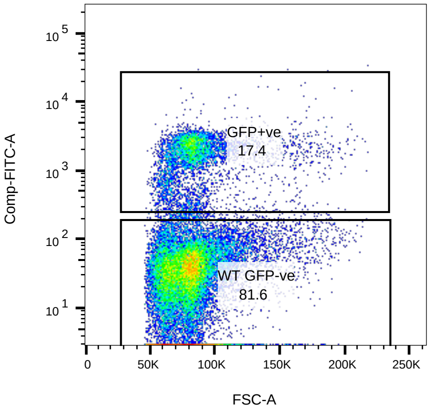

cw171221 UBCCre R26Adar1_BM M A1 Za 121_037.fcs
Lymphocytes
30205

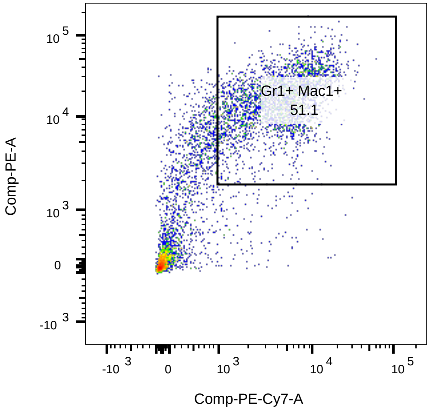

cw171221 UBCCre R26Adar1_BM M A1 Za 121_037.fcs
GFP+ve
5259

Bone Marrow
Myeloid cells
Antibodies:
Gr-1 PE-Cy7
CD11b PE
F4/80 APC

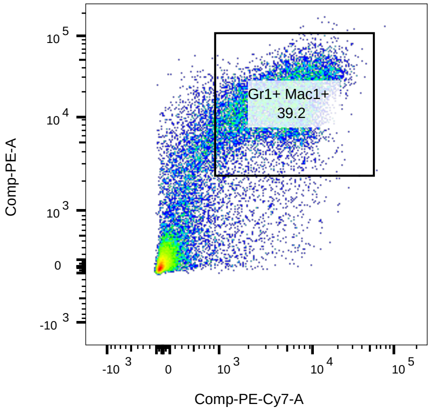

cw171221 UBCCre R26Adar1_BM M A1 Za 121_037.fcs
WT GFP-ve
24643

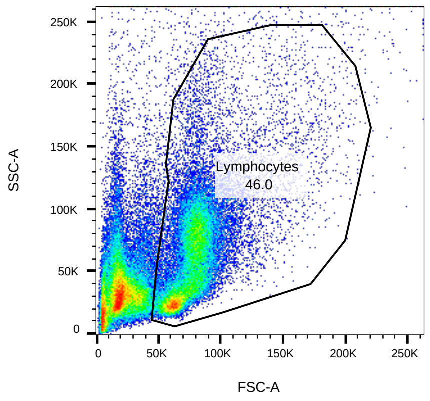

cw171221 UBCCre R26Adar1_BM B A1 Za 121_057.fcs
Ungated
66250

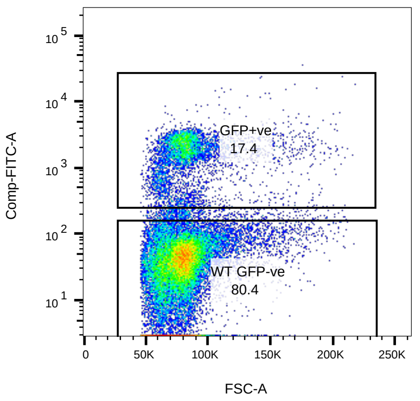

cw171221 UBCCre R26Adar1_BM B A1 Za 121_057.fcs
Lymphocytes
30478

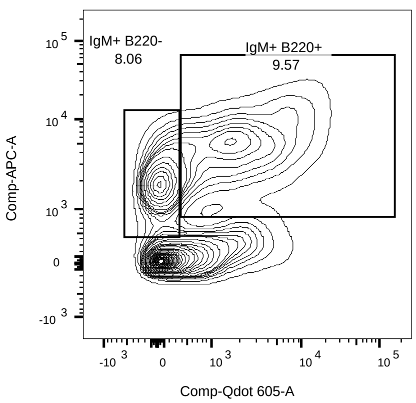

cw171221 UBCCre R26Adar1_BM B A1 Za 121_057.fcs
GFP+ve
5296

Bone Marrow
B cells
Antibodies:
B220 APC
IgM biotin - Streptavidin BV605 (secondary)

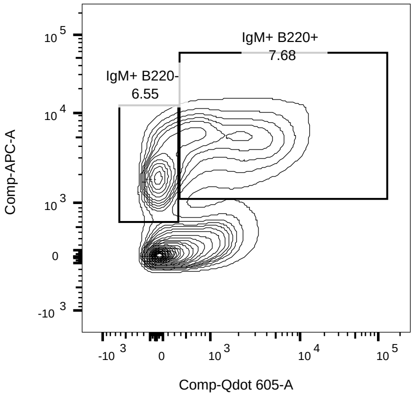

cw171221 UBCCre R26Adar1_BM B A1 Za 121_057.fcs
WT GFP-ve
24519

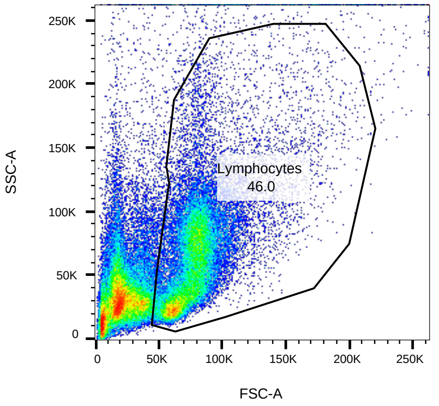

cw171221 UBCCre R26Adar1_BM T A1 Za 121_067.fcs
Ungated
66465

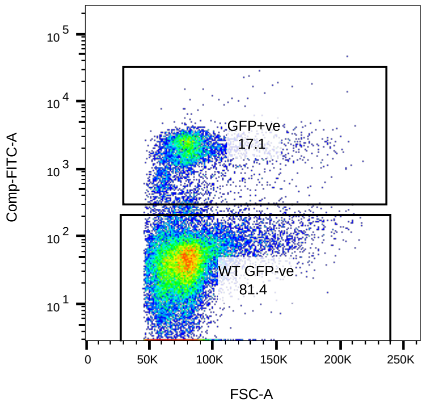

cw171221 UBCCre R26Adar1_BM T A1 Za 121_067.fcs
Lymphocytes
30578

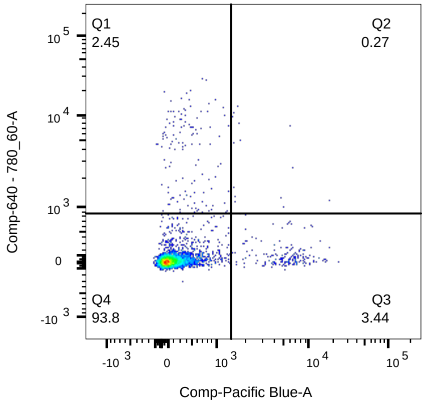

cw171221 UBCCre R26Adar1_BM T A1 Za 121_067.fcs
GFP+ve
5235

Bone Marrow
T cells
Antibodies:
CD4 eF450
CD8 APC-eF780

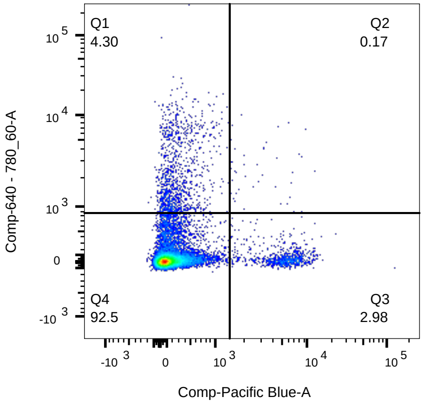

cw171221 UBCCre R26Adar1_BM T A1 Za 121_067.fcs
WT GFP-ve
24880

Spleen
Antibodies:
Gr-1 PE-Cy7
CD11b PE
F4/80 APC
B220 APC-ef780
CD4 eF450
CD8 PerCP-Cy5.5

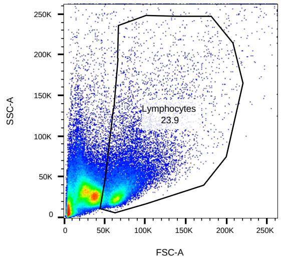

cw171221 UBCCre R26Adar1_Spl A1 Za 121_027.fcs
Ungated
130907

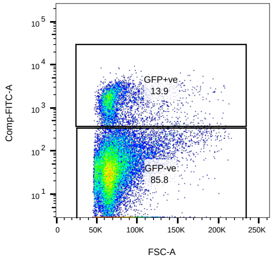

cw171221 UBCCre R26Adar1_Spl A1 Za 121_027.fcs
Lymphocytes
31272

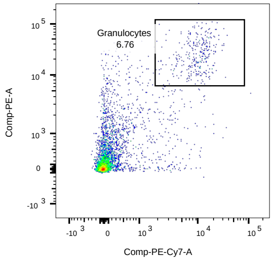

cw171221 UBCCre R26Adar1_Spl A1 Za 121_027.fcs
GFP+ve
4332

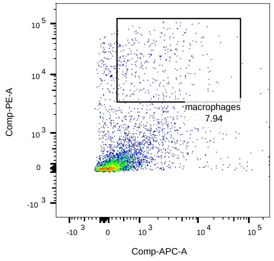

cw171221 UBCCre R26Adar1_Spl A1 Za 121_027.fcs
GFP+ve
4332

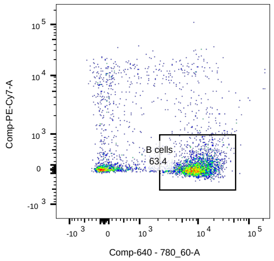

cw171221 UBCCre R26Adar1_Spl A1 Za 121_027.fcs
GFP+ve
4332

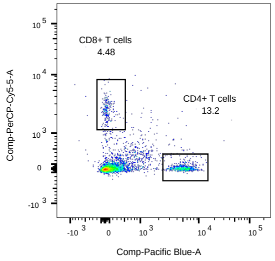

cw171221 UBCCre R26Adar1_Spl A1 Za 121_027.fcs
GFP+ve
4332

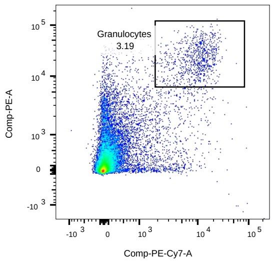

cw171221 UBCCre R26Adar1_Spl A1 Za 121_027.fcs
GFP-ve
26827

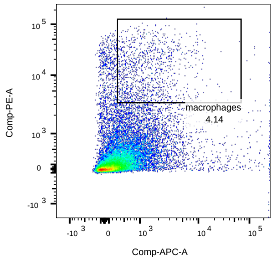

cw171221 UBCCre R26Adar1_Spl A1 Za 121_027.fcs
GFP-ve
26827

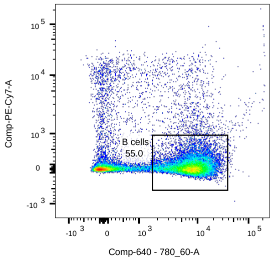

cw171221 UBCCre R26Adar1_Spl A1 Za 121_027.fcs
GFP-ve
26827

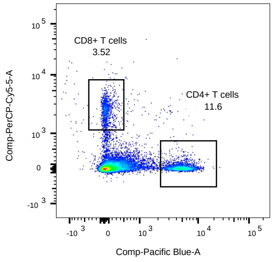

cw171221 UBCCre R26Adar1_Spl A1 Za 121_027.fcs
GFP-ve
26827

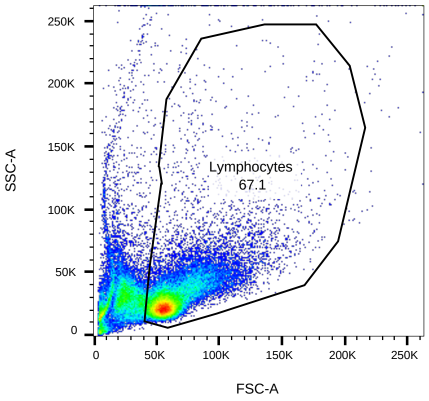

cw171221 UBCCre R26Adar1_Thy A1 Za 121_077.fcs
Ungated
52052

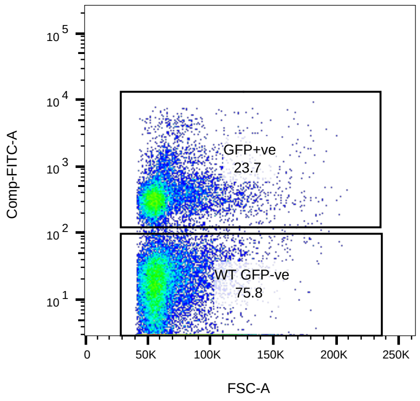

cw171221 UBCCre R26Adar1_Thy A1 Za 121_077.fcs
Lymphocytes
34912

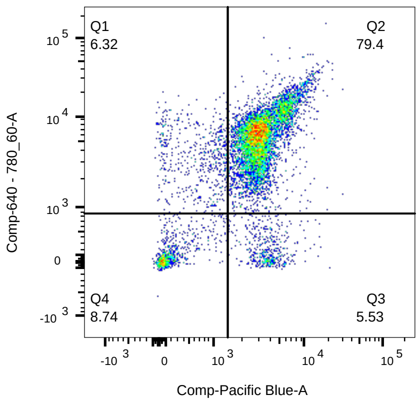

cw171221 UBCCre R26Adar1_Thy A1 Za 121_077.fcs
GFP+ve
8277

Thymus
T cells
Antibodies:
CD4 eF450
CD8 APC-eF780

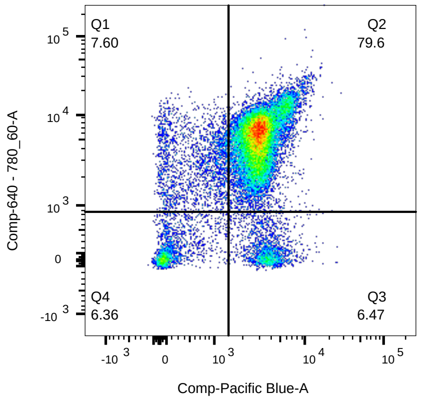

cw171221 UBCCre R26Adar1_Thy A1 Za 121_077.fcs
WT GFP-ve
26475

Peripheral Blood (following RBC lysis)
Antibodies:
Gr-1 PE-Cy7
CD11b PE
F4/80 APC
B220 APC-ef780
CD4 eF450
CD8 PerCP-Cy5.5

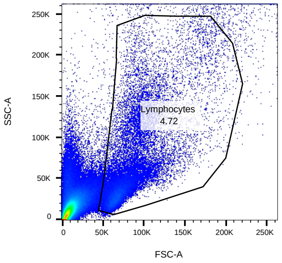

cw171221 UBCCre R26Adar1_PB A1 Za 121_017.fcs
Ungated
6.48e5

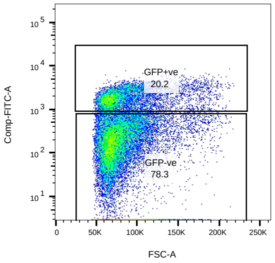

cw171221 UBCCre R26Adar1_PB A1 Za 121_017.fcs
Lymphocytes
30551

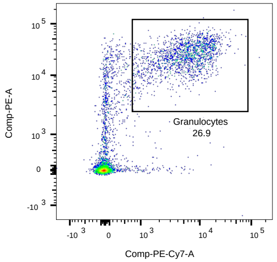

cw171221 UBCCre R26Adar1_PB A1 Za 121_017.fcs
GFP+ve
6179

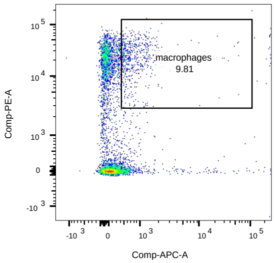

cw171221 UBCCre R26Adar1_PB A1 Za 121_017.fcs
GFP+ve
6179

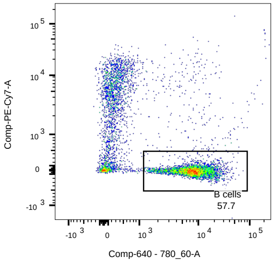

cw171221 UBCCre R26Adar1_PB A1 Za 121_017.fcs
GFP+ve
6179

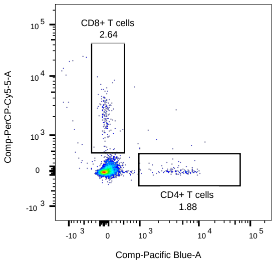

cw171221 UBCCre R26Adar1_PB A1 Za 121_017.fcs
GFP+ve
6179

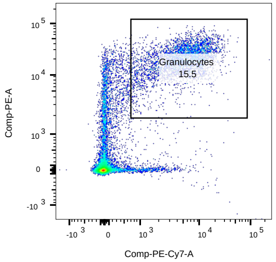

cw171221 UBCCre R26Adar1_PB A1 Za 121_017.fcs
GFP-ve
23913

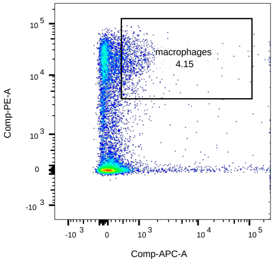

cw171221 UBCCre R26Adar1_PB A1 Za 121_017.fcs
GFP-ve
23913

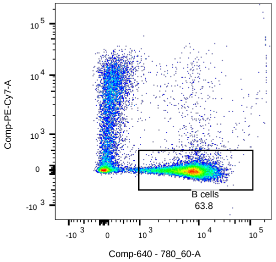

cw171221 UBCCre R26Adar1_PB A1 Za 121_017.fcs
GFP-ve
23913

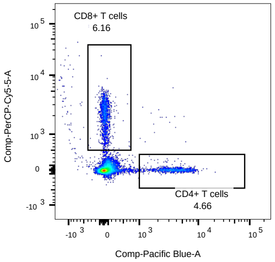

cw171221 UBCCre R26Adar1_PB A1 Za 121_017.fcs
GFP-ve
23913

Supplementary Table 1. Genotyping primers used in this study.

Genotyping oligonucleotide list

Targeted genotyping allele	Oligonucleotide sequence (5'-3')	Product size (base pairs)
Ubc-CreER	GAC GTC ACC CGT TCT GTT G (25285-UBC-A)	WT = 324bp<br>KI = 475bp
	CTA GGC CAC AGA ATT GAA AGA TCT (oMIR7338)	
	GTA GGT GGA AAT TCT AGC ATC ATC C (oMIR7339)	
	AGG CAA ATT TTG GTG TAC GG (oMIR79074-Cre-A)	
R26-Adar1 alleles	GCC TCC TGG CTT CTG AGG ACC G (R26F2)	WT = 200bp<br>KI = 394bp
	TCT GTG GGA AGT CTT GTC CCT CC (R26R2)	
	GGA AAG TCC CTA TTG GCG TTA (CAG enhancer Rev)	
R26-eYFP	AAA GTC GCT CTG AGT TGT TAT (R26eYFP_1)	WT = 500bp<br>KI = 250bp
	GCG AAG AGT TTG TCC TCA ACC (R26eYFP_2)	
	GGA GCG GGA GAA ATG GAT ATG (R26eYFP_3)	
Osx1-Cre	GCC AGG CAG GTG CCT GGA CAT (TGCK)	Tg = 450bp
	CTC TTC ATG AGG AGG ACC CT (Osx-10)	
*Trp53^fl/fl^*	CAC AAA AAC AGG TTA AAC CCA G (p53F2-10 1F)	WT = 288bp<br>Floxed = 370bp
	AGC ACA TAG GAG GCA GAG AC (p53F2-10 1R)	
*Rb1^fl/fl^*	CTC TAG ATC CTC TCA TTC TTC (Rb 5'loxP)	WT = 260bp<br>Floxed = 308bp
	CCT TGA CCA TAG CCC AGC AC (Rb 3'loxP)	
LoxP-flanked Stop cassette deletion at *Rosa* locus	TGG GCA ACG TGC TGG TTA TT (Cag For1)	WT = 302bp<br>Recombinant ADAR1 = 431bp<br>Recombinant ADAR1p110 = 485bp
	ATC AGG ACA TAG CGT TGG CT (Neo Rev1)	
	CCA GTG TCC TGG GAG GAA TG (*R26-Adar1* Rev 1)	
	AGG GAA TGA GGG GCT TCT AGT (*R26-Adar1p110* REV)	
LoxP-flanked Stop cassette knock-out of p53	AAG GGG TAT GAG GGA CAA GG (*P53*F2-10 10F)	WT = 288bp<br>P53 knock-out = 431bp
	GAA GAC AGA AAA GGG GAG GG (*P53*F2-10 10R)	
	CAC AAA AAC AGG TTA AAC CCA G (*P53*F2-1F)	WT = 548bp<br>P53 knock-out = 370bp
	AGC ACA TAG GAG GCA GAG AC (*P53*F2-1R)	

Supplementary Table 2. Flow cytometry antibodies, dye conjugates, antibody clone and source used in this study.

Antibody (clone)	Conjugate	Final Dilution	Catalogue number	Supplier
**Gr1**	PE-Cy7	1:1000	25-5931-82	Life Technologies Australia Pty Ltd/Thermo Fisher
**CD11b/Mac1**	PE	1:400	12-0112-83	Life Technologies Australia Pty Ltd/Thermo Fisher
**F4/80**	APC	1:250	20-4801-U100	Tonbo Biosciences
**B220**	APC-eFlour780	1:200	17-0452083	Life Technologies Australia Pty Ltd/Thermo Fisher
**CD4**	eFlour-450	1:400	48-0042-82	Life Technologies Australia Pty Ltd/Thermo Fisher
**CD8a**	PerCP-Cy5.5	1:200	45-0081-82	Life Technologies Australia Pty Ltd/Thermo Fisher
**Ter119**	PE	1:400	12-5921-83	Life Technologies Australia Pty Ltd/Thermo Fisher
**CD71**	APC	1:400	17-0711-82	Life Technologies Australia Pty Ltd/Thermo Fisher
**CD44**	PE-Cy7	1:1200	25-0441-82	eBioscience
**CD43**	PE	1:200	553271	BD Pharmingen
**B220**	APC	1:400	17-0452-83	Life Technologies Australia Pty Ltd/Thermo Fisher
**CD19**	PerCP-Cy5.5	1:150	45-0193-82	Life Technologies Australia Pty Ltd/Thermo Fisher
**CD8a**	APC-eFlour780	1:400	47-0081-82	Life Technologies Australia Pty Ltd/Thermo Fisher
**TCRβ**	PE	1:400	12-5961-83	Life Technologies Australia Pty Ltd/Thermo Fisher
**CD25**	PE	1:400	12-0251-83	Life Technologies Australia Pty Ltd/Thermo Fisher
**CD44**	APC	1:400	17-0441-83	Life Technologies Australia Pty Ltd/Thermo Fisher
**Sca-1**	APC	1:200	17-5981-82	Life Technologies Australia Pty Ltd/Thermo Fisher
**c-Kit**	APC-eFlour780	1:200	47-1171-82	Life Technologies Australia Pty Ltd/Thermo Fisher
**CD150**	PE	1:100	115904	Biolegend
**CD105**	PE-Cy7	1:400	120409	Biolegend

**FcγRII (CD16/32)**	PerCP-Cy5.5	1:300	45-0161-82	Life Technologies Australia Pty Ltd/Thermo Fisher
**CD41**	eFlour-450	1:200	48-0411-82	Life Technologies Australia Pty Ltd/Thermo Fisher
**Sca-1**	PE-Cy7	1:100	25-5981-82	Life Technologies Australia Pty Ltd/Thermo Fisher
**CD34**	eFlour-660	1:25	50-0341-82	Life Technologies Australia Pty Ltd/Thermo Fisher
**CD135 (Flt3)**	PE	1:100	12-1351-82	Life Technologies Australia Pty Ltd/Thermo Fisher
**FcγRII (CD16/32)**	eFlour-450	1:400	48-0161-82	Life Technologies Australia Pty Ltd/Thermo Fisher
**CD2**	Biotin	1:400	13-0021-85	Life Technologies Australia Pty Ltd/Thermo Fisher
**CD3e**	Biotin	1:400	30-0031-U500	Tonbo Biosciences
**CD5**	Biotin	1:400	13-0051-85	Life Technologies Australia Pty Ltd/Thermo Fisher
**CD4**	Biotin	1:400	13-0041-85	Life Technologies Australia Pty Ltd/Thermo Fisher
**CD8a**	Biotin	1:400	13-0081-85	Life Technologies Australia Pty Ltd/Thermo Fisher
**CD11b**	Biotin	1:400	13-0112-85	Life Technologies Australia Pty Ltd/Thermo Fisher
**Ter119**	Biotin	1:400	30-5921-U500	Tonbo Biosciences
**Gr-1**	Biotin	1:400	13-5931-85	Life Technologies Australia Pty Ltd/Thermo Fisher
**B220**	Biotin	1:400	13-0452-85	Life Technologies Australia Pty Ltd/Thermo Fisher
**CD45RB**	Biotin	1:400	13-0455-81	Life Technologies Australia Pty Ltd/Thermo Fisher
**IgM**	Biotin	1:400	13-5790-82	Life Technologies Australia Pty Ltd/Thermo Fisher
**Streptavidin**	BV605	1:400	563260	BD Biosciences
